# [^18^F]FET uptake in diffuse lower-grade IDH-mutant glioma correlates with glioma-related epilepsy

**DOI:** 10.1007/s11060-026-05815-w

**Published:** 2026-10-01

**Authors:** Zeynep Özdemir, Wolfgang Roll, Walter Stummer, Stjepana Kovac, Michael Müther

**Affiliations:** 1 Department of Neurosurgery, University Hospital of Münster, Albert-Schweitzer-Campus 1, A1 D-48149 Münster, Germany; 2 Department of Nuclear Medicine, University Hospital Münster, Münster, Germany; 3 Department of Neurology, University Hospital Münster, Münster, Germany

**Keywords:** DLGG, Glioma-related epilepsy, Glioma, FET PET, Seizures

## Abstract

**Purpose:**

Diffuse lower-grade glioma (DLGG), i.e. WHO grade 2 and 3 tumors, frequently manifests with epileptic seizures, significantly affecting quality of life. While [^18^F]FET PET is routinely used to assess glioma metabolism, differences between patients with and without glioma-related epilepsy have not yet been assessed systematically. The objective of this study was to assess whether preoperative [^18^F]FET PET uptake is associated with epileptic seizures at initial presentation in patients with DLGG.

**Methods:**

This retrospective study included 121 patients with DLGG with preoperative [^18^F]FET PET (2015–2025). [^18^F]FET uptake parameters were determined in patients with measurable disease according to PET RANO 1.0 criteria. Seizure status was assessed exclusively for seizures occurring before surgery. Statistical analyses included Fisher’s exact test, Mann-Whitney U test, and multivariable logistic regression.

**Results:**

Seventy-eight patients (64%) demonstrated measurable disease in [^18^F]FET, of whom 57 (73%) presented with epileptic seizures compared to 17 (40%) in the no-uptake group (*p* < 0.001). In the measurable disease group, those with seizures exhibited significantly higher median TBR_max_ (3.02; IQR 2.35–4.00) and TBR_mean_ (2.2; IQR 1.8–2.5) values than those without seizures (TBR_max_ 2.4; IQR 2.00–3.16, TBR_mean_ 1.9; IQR 1.7–2.2). The tumor location did not account for the observed association between [^18^F]FET uptake and seizures (*p* > 0.05). In multivariable analyses, [^18^F]FET uptake remained independently associated with preoperative seizures (adjusted OR 2.86, 95% CI 1.18–7.07; *p* = 0.020).

**Conclusion:**

These findings demonstrate an association between [^18^F]FET uptake and glioma-related epilepsy in DLGG. Further research on amino acid transporter expression and glutamatergic pathways may elucidate underlying mechanisms.

## Introduction

The most common initial clinical manifestation of IDH-mutant WHO grades 2 and 3 diffuse lower-grade glioma (DLGG) is an epileptic seizure, occurring in up to 75% of cases [1]. Tumor location is a critical factor regarding the development of seizures [2]. Specifically, tumors involving the frontal and temporal lobes have been associated with an increased risk. Glioma-related epilepsy (GRE) has a negative impact on quality of life. Long-term seizures and long-term use of antiseizure medication (ASM) are associated with cognitive impairment [3].

Key mechanisms implicated in GRE include elevated glutamate levels produced by glioma cells which promote tumor proliferation via activation of NMDA and AMPA receptors, resulting in neuronal hyperexcitability, as well as the upregulation of the mTOR-signaling pathway which is induced by increased D-2-hydroxyglutarate (2-HG) production in IDH-mutant glioma [4–6].

Amino acid PET is routinely employed as a diagnostic adjunct in the treatment of glioma, both for surgical planning and response assessment during adjuvant treatment [7–11]. Radio-labeled amino acids are selectively taken up in glioma cells via L-type amino acid transporter 1 (LAT1) which is highly upregulated in glioma cells [12–14]. Tumor heterogeneity can be assessed more reliably with amino acid PET than MRI as tracer uptake does not only rely on the disruption of the blood brain barrier [15]. Transient increase of [^18^F]FET uptake during a symptomatic epileptic seizures has been described [16].

Although clinical evidence suggests changes in amino acid tracer uptake during seizure activity, general differences in [^18^F]FET uptake of DLGG in patients with and without GRE have not yet been assessed in a larger cohort. The aim of this retrospective study is to elucidate a potential correlation between amino acid uptake, assessed by [^18^F]FET PET and the presence of seizures at initial presentation in DLGG patients.

## Methods

This retrospective study identified patients with preoperative [^18^F]FET PET imaging and subsequent neuropathological confirmation of primary grade 2 and 3 DLGG from 2015 to 2025 using the single-center brain tumor database of University Hospital Münster, Münster, Germany. Data extraction and analysis were performed between July 2025 and January 2026. Diagnoses prior to 2021 were adjusted in adherence to the WHO CNS 2021 classification. Patients with non-lobar tumors and [^18^F]FET PET imaging older than six months prior to surgery were excluded. All identified patients were assessed for the presence or absence of epileptic seizures at the time of [^18^F]FET PET imaging. Seizure type was retrospectively determined from the initial neurological assessment, including documentation provided by the referring physicians. EEG data were not available across all patients and have therefore, not been included in this study. The development of epileptic seizures during the subsequent course of treatment, including surgery and potential adjuvant chemoradiation, was not assessed. [^18^F]FET PET was conducted in accordance with procedural recommendations [17]. In all acquired [^18^F]FET PET, tumor to brain ratios (TBR) and PET-positive tumor volume were calculated. In order to assess [^18^F]FET PET in glioma in a standardized and reproducible manner, PET RANO 1.0 criteria were applied [9]. Accordingly, lesions with TBR_max_ > 1.6 and a PET-positive tumor volume ≥ 0.5mL were defined as measurable disease and will be referred to as the measurable disease group in the present study [9]. No measurable disease and non-measurable disease, i.e. TBR_max_ < 1.6 or PET-positive tumor volume < 0.5mL, will be referred to as the no-uptake group [9]. The spatial distribution of [^18^F]FET measurable disease was additionally assessed in relation to the FLAIR-positive tumor volume, including potential extension into adjacent cortical or other perilesional regions. [^18^F]FET PET examinations were performed using either a 3-T PET/MRI system (Biograph mMR; Siemens Healthcare GmbH, Erlangen, Germany) or a PET/CT system (Biograph mCT; Siemens Healthcare GmbH). [¹⁸F]FET was synthesized onsite and administered at 2.5 MBq/kg body weight after fasting for at least 6 h. PET acquisition was performed dynamically for 40 min from tracer injection or as a static acquisition from 20 min post-injection. Fisher’s exact test was used to compare categorical variables. To test for normal distribution of variables, Shapiro-Wilk and Kolmogorov-Smirnov tests were used. Continuous variables were compared using the Mann-Whitney U test as a nonparametric test. A multiple variable logistic regression model was used to assess whether [^18^F]FET uptake was independently associated with seizure occurrence at presentation. The model was adjusted for contrast enhancement, tumor side, tumor type, tumor location and MRI-derived tumor volume. Model performance was assessed by evaluating goodness of fit, calibration, and discrimination. Statistical analyses were performed using GraphPad Prism (Version 10.1.1, GraphPad Software, Boston, Massachusetts USA) and R version 4.5.3 (R Foundation for statistical computing, Vienna, Austria). Statistical significance was set at *p* < 0.05. Ethics approval was obtained from the Ethics Committee of Westfalen-Lippe, Münster, Germany (reference number 2020-091-f-S). The study was conducted in accordance with the 1964 Declaration of Helsinki and its later amendments. Due to the retrospective nature of the study and the use of routinely collected clinical data, the requirement for written informed consent was waived by the local ethics committee.

## Results

**Fig. 1:**
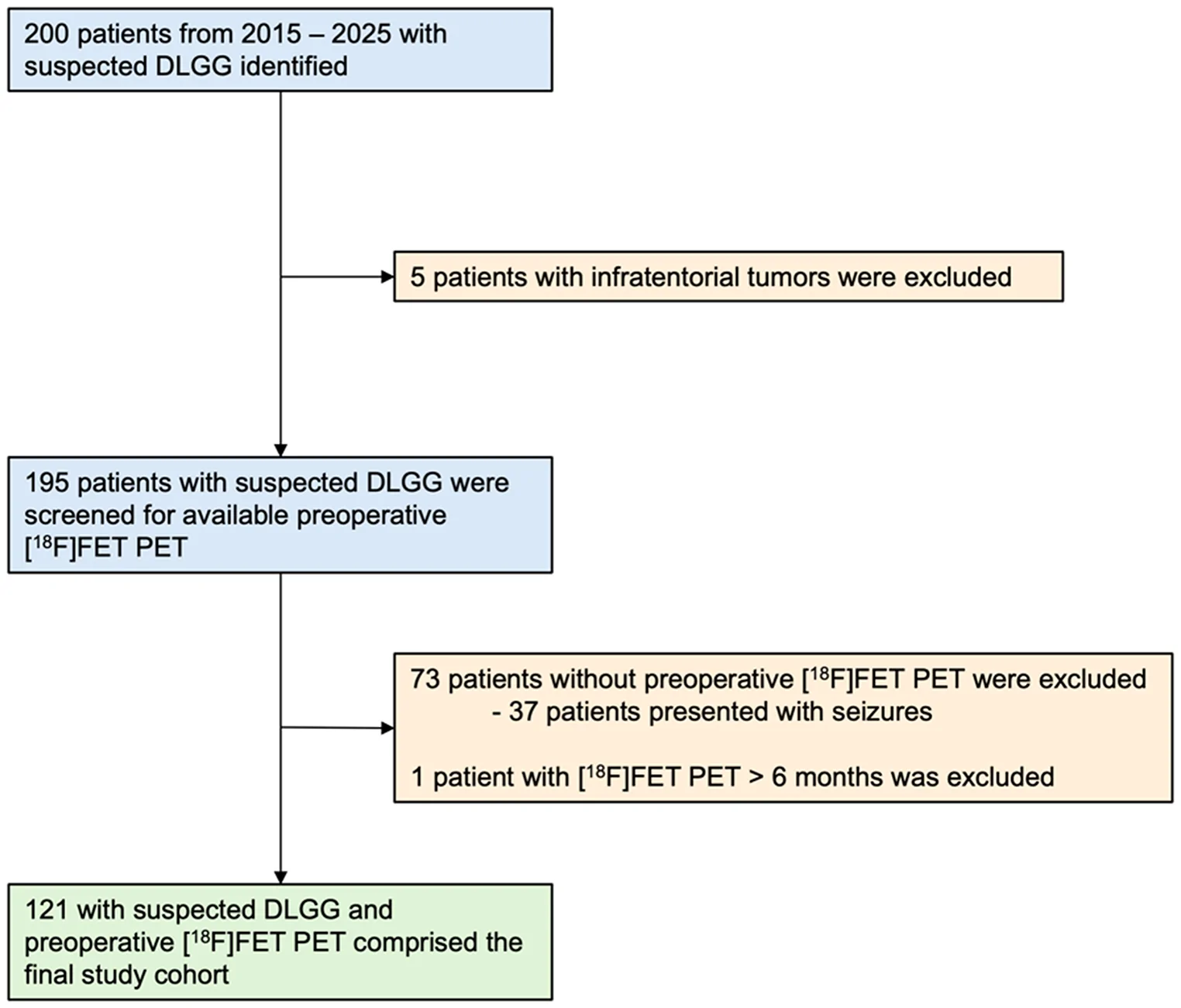
Study flow chart

**Fig. 2:**
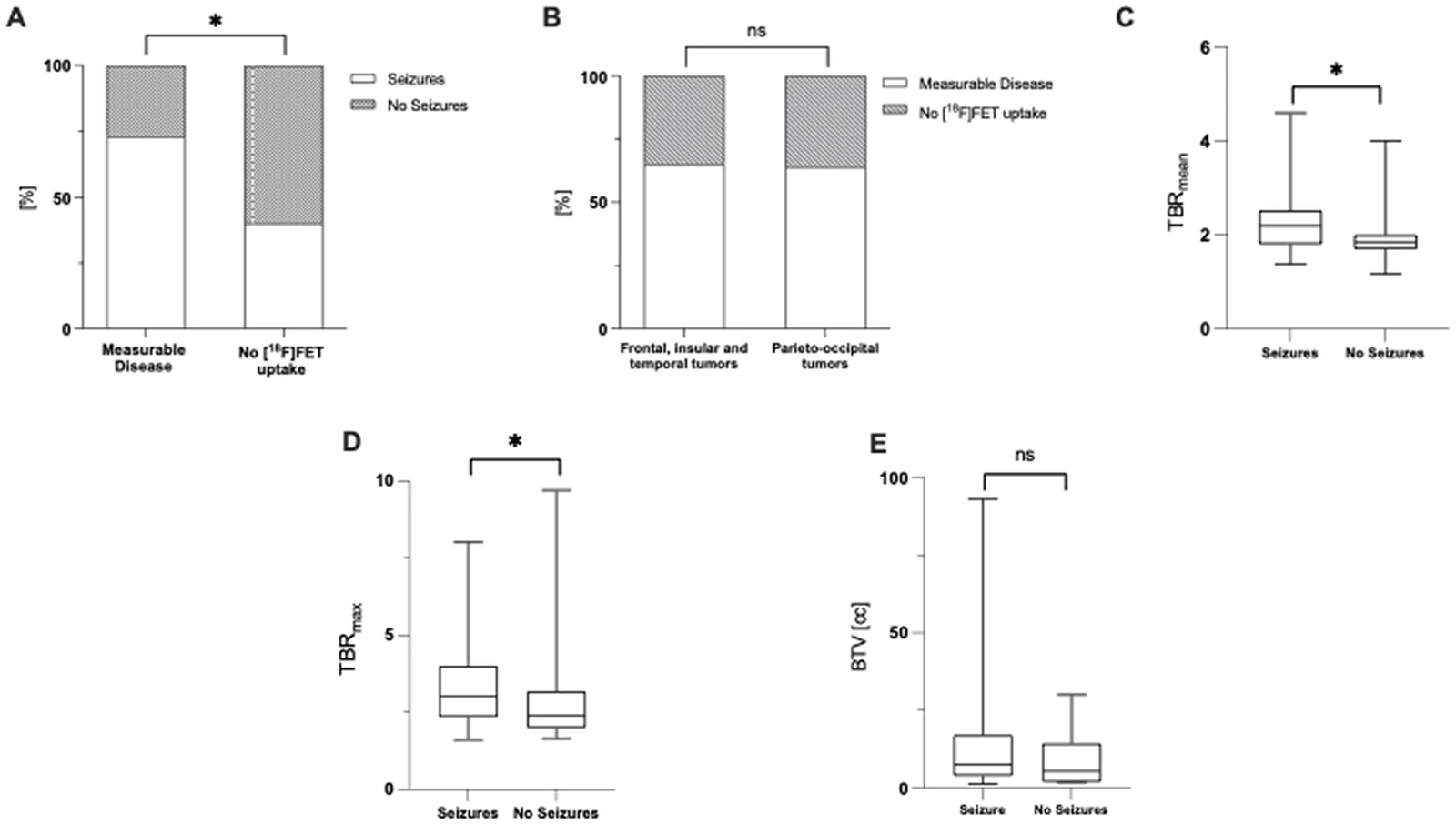
**(A)** Fisher’s exact test demonstrated a significant difference (*p* < 0.001) in the prevalence of epileptic seizures in patients with and without [^18^F]FET uptake with an odds ratio of 4.15 (95% CI 1.90–8.94). **(B)** Fisher’s exact test did not show a statistically significant difference (*p* > 0.05) regarding the prevalence of [^18^F]FET uptake in frontal, temporal and insular tumors compared with parieto-occipital tumors. **(C)** TBR_mean_ in the seizure group was also significantly higher (*p* = 0.04) with a median TBR_mean_ in the seizure group of 2.2 (IQR 1.8–2.5) compared to 1.9 (IQR 1.7–2.2). **(D)** Mann-Whitney U test showed significant difference in TBR_max_ values between the seizure and no-seizure group (*p* = 0.03) with a median TBR_max_ of 3.02 (IQR 2.35–4.00) in the seizure group compared to 2.4 (IQR 2.00–3.16) in the no-seizure group. Boxes indicate 25% and 75% quartiles, whiskers minimum and maximum values, horizontal lines the median. **(E)** Mann-Whitney U test did not show a significant difference (*p* > 0.05) regarding BTV between groups

**Fig. 3:**
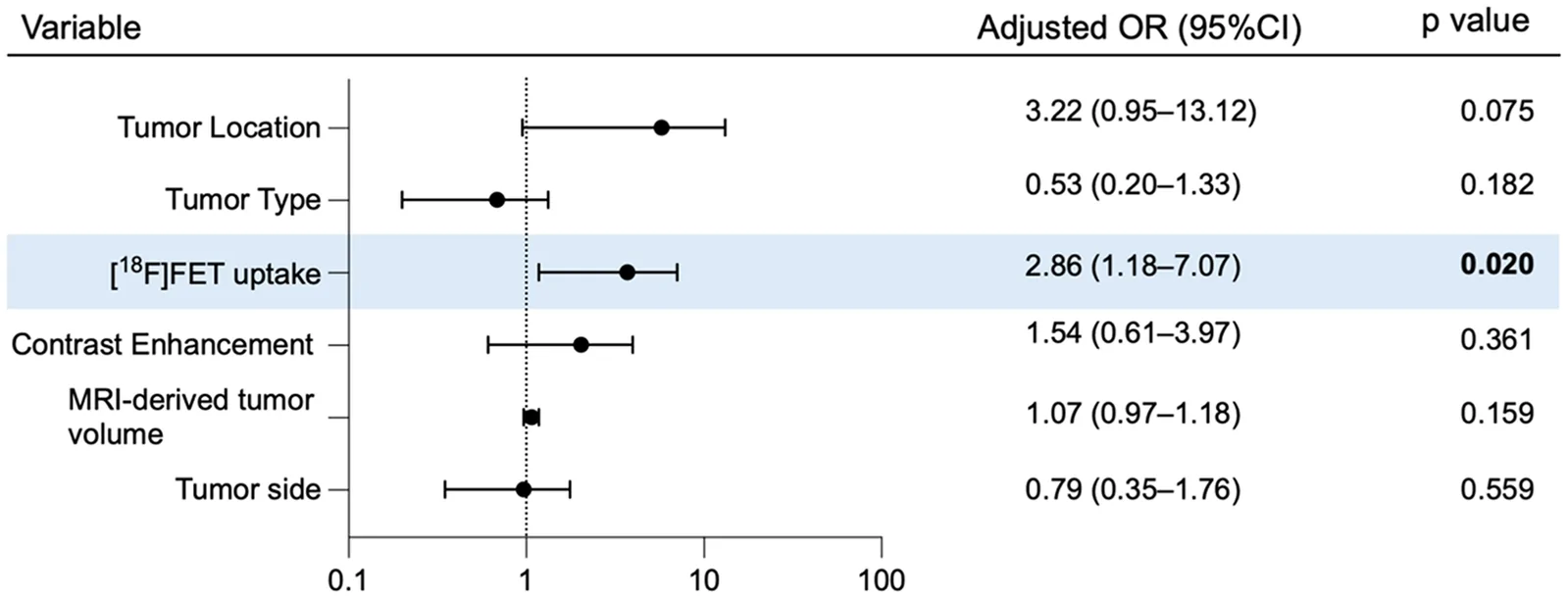
Forest plot of multivariable logistic regression analysis of factors associated with seizure presentation in supratentorial DLGG. Adjusted ORs with 95% CIs are displayed on a logarithmic scale. The vertical dashed line indicates OR = 1. The model was adjusted for contrast enhancement, tumor side, tumor location, tumor type, and tumor volume

**Fig. 4:**
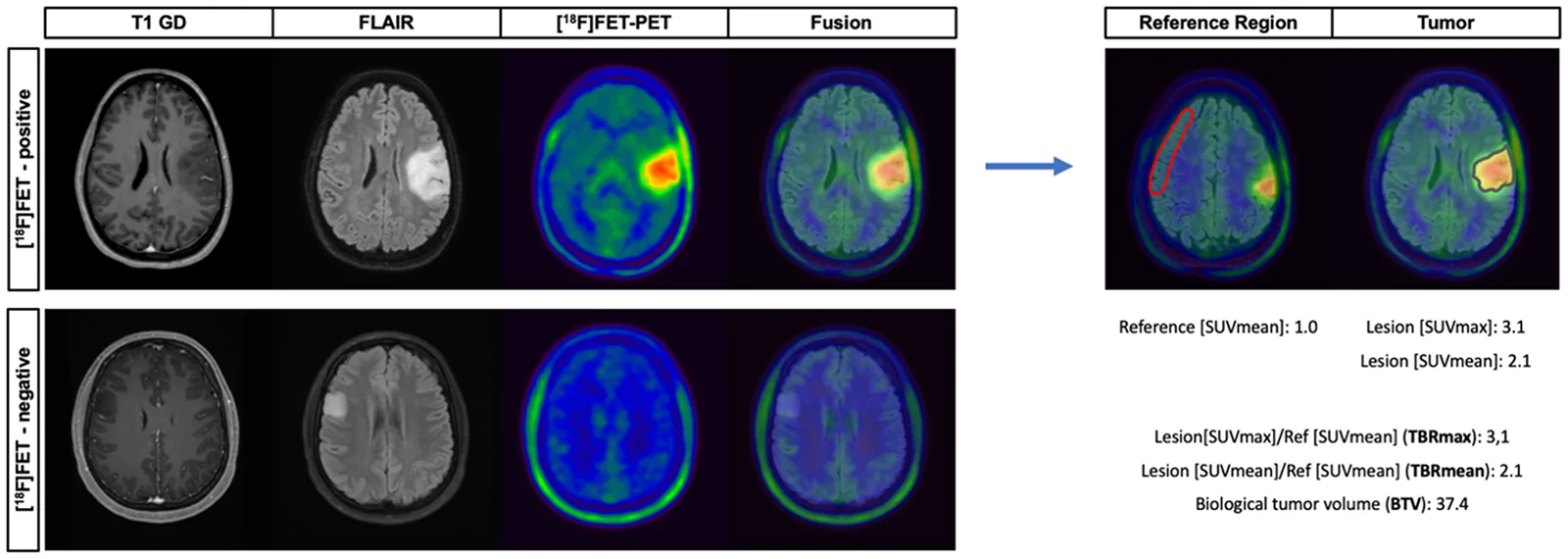
[^18^F]FET PET and MRI in DLGG. Contrast enhanced T1-weighted MRI, FLAIR and [^18^F]FET PET image fusion in a patient presenting with seizures at initial diagnosis (*top row*) and a patient without seizures at initial diagnosis (*bottom row*)

A total of 200 patients with suspected DLGG were identified. Of these, five patients with infratentorial tumors, 73 without preoperative [^18^F]FET PET, and one patient with preoperative [^18^F]FET PET performed more than 6 months before surgery were excluded (Fig. 1), resulting in a final study cohort of 121 (100%) patients with IDH-mutant DLGG with preoperative [^18^F]FET PET. In all included patients, [^18^F]FET PET was performed as part of routine preoperative diagnostic work-up. Patients without preoperative [^18^F]FET PET predominantly originated from earlier years of the study period when this had not been established as part of the routine preoperative work-up. Baseline characteristics including seizure characteristics are summarized in Table 1. Measurable disease in preoperative [^18^F]FET PET was observed in 78 patients (64%). PET-positive volumes were confined to FLAIR-positive tumor volume in all of these cases and did not extend to perilesional cortex. The no-uptake group comprised 43 (36%) patients in total, including 14 patients (12%) with non-measurable disease and 29 patients (24%) with no measurable disease. Of those with [^18^F]FET uptake, 57 patients (73%) presented with epileptic seizures, while the remaining 21 patients (27%) did not develop preoperative epileptic seizures. In the no-uptake group, epileptic seizures were the initial clinical manifestation in 17 patients (40%), whereas 26 patients (60%) did not develop epileptic seizures preoperatively. Fisher’s exact test demonstrated a significant difference in the prevalence of epileptic seizures between patients with and without measurable disease (*p* < 0.001), with an odds ratio of 4.15 (95% CI 1.90–8.94). Within the measurable disease group, Mann-Whitney U test was performed to compare TBR_max,_ TBR_mean_ and biological tumor volume (BTV) between the seizure and no-seizure group. There was a significant difference (*p* = 0.04) between TBR_mean_ values with a median TBR_mean_ in the seizure group of 2.2 (IQR 1.8–2.5) compared to 1.9 (IQR 1.7–2.2) in the no-seizure group (Table 2; Fig. 2). The median TBR_max_ value in the seizure group was also significantly (*p* = 0.03) higher with 3.02 (IQR 2.35–4.00) compared to 2.4 (IQR 2.00–3.16) in the no-seizure group. There was no significant difference (*p* > 0.05) regarding BTV between the groups (see Fig. 2). In multivariable logistic regression adjusting for contrast enhancement, tumor side, MRI-derived tumor volume, tumor type, and tumor location, [^18^F]FET uptake remained independently associated with seizure presentation (adjusted OR 2.86, 95% CI 1.18–7.07; *p* = 0.020, Fig. 3). The model showed moderate discrimination (AUC 0.728, 95% CI 0.635–0.822) and no evidence of lack of fit (Hosmer–Lemeshow test, χ²=4.32, *p* = 0.827). The median interval between first epileptic seizure and [^18^F]FET PET acquisition was 13 days (IQR 8.25–25.75; Table 2). To assess whether [^18^F]FET uptake may have been influenced by temporal proximity to the index seizure, the association between seizure-to-PET interval and quantitative [^18^F]FET uptake was examined among patients presenting with seizures. No correlation was observed between seizure-to-PET interval and either TBR_max_ (Spearman’s ρ = −0.058, *p* = 0.69) or TBR_mean_ (ρ = −0.043, *p* = 0.79). In an additional sensitivity analysis using a 14-day cutoff, TBR_max_ did not differ between patients undergoing [^18^F]FET-PET < 14 days versus ≥ 14 days after seizure onset (median 3.11 (IQR 2.39–3.85) vs. 3.00 (IQR 2.26–4.40), *p* = 0.86). Similarly, TBR_mean_ was comparable between the two groups (2.14 (IQR 1.70–2.50) vs. 2.10 (IQR 1.80–2.50), *p* = 0.94). Within the measurable disease group, there was no statistically significant difference (*p* > 0.05) between the prevalence of epileptic seizures in oligodendrogliomas (*n* = 25; 81%) and astrocytomas (*n* = 32; 68%). 104 tumors (86%) were located in the frontal, temporal, or insular lobes, whereas 17 (14%) were located in the parietal or occipital lobes. Fisher’s exact test did not show a significant difference regarding the prevalence of epileptic seizures (*p* > 0.05) between frontal/temporal/insular (67/104, 64%) and parieto-occipital tumors (11/17, 65%, Fig. 2). See Fig. 4 for illustrative examples of DLGG with and without [^18^F]FET uptake.

**Table 1:** Baseline characteristics of the study cohort

Total study cohort	121 (100%)
Epileptic seizures at initial presentation	74 (61%)
Generalized seizures	32 (44%)
Focal seizures	27 (36%)
Unclassified	15 (20%)
Initiation of antiseizure medication	72 (97%)
Levetiracetam	64 (89%)
Lacosamide	6 (8%)
Lamotrigine	2 (3%)
Uncontrollable seizures	0
Status epilepticus	0
Median interval from first epileptic seizure to acquisition of [^18^F]FET PET imaging (days)	13 (IQR 8.25–25.75)
Tumor location	*p* > 0.05
Frontal, insular and temporal tumors Measurable Disease No [^18^F]FET uptake	104(86%) *67 (64%)* *37 (36%)*
Parieto-occipital tumors Measurable Disease No [^18^F]FET uptake	17 (14%) *11 (65%)* *6 (35%)*

**Table 2:** [^18^F]FET PET characteristics between the cohorts

	Seizures	No Seizures	*p*-value
Age (years)	40.5 (IQR 32–50)	40.0 (IQR 30–49)	>0.05
Tumor Type			*>0.05*
Astrocytoma	47 (56%)	37 (44%)	
Oligodendroglioma	27 (73%)	10 (27%)	
WHO Grade			*>0.05*
2	71 (61%)	46 (39%)	
3	3 (75%)	1 (25%)	
Contrast enhancing tumor	30 (73%)	11 (27%)	*> 0.05*
[^18^F]FET uptake and seizure prevalence			*0.0004*
Measurable Disease	57 (73%)	21 (27%)	
No [^18^F]FET uptake	17 (40%)	26 (60%)	
Median TBR_max_ in measurable disease	3.02 (IQR 2.35–4.00)	2.4 (IQR 2.00–3.16)	*0.03*
Median TBR_mean_ in measurable disease	2.2 (IQR 1.8–2.5)	1.9 (IQR 1.7–2.2)	*0.04*
Median BTV	7.6 (IQR 3.9–17.2)	5.4 (IQR 1.9–14.3)	*> 0.05*

## Discussion

The aim of the present study was to explore differences in metabolic activity on preoperatively acquired [^18^F]FET PET imaging between patients with and without epileptic seizures as the first clinical manifestation. Our results demonstrate a significant association between preoperative amino-acid uptake and epileptic seizures as initial clinical presentation. TBR_max_ and TBR_mean_ values in the seizure group were significantly higher. Multivariable regression analysis further supported these findings demonstrating that [^18^F]FET uptake remained independently associated with seizure occurrence at initial presentation after adjustment for contrast enhancement, tumor side, tumor type, tumor location and tumor volume. However, the limited number of WHO grade 3 tumors, precluded meaningful adjustment for tumor grade. Although BTV was slightly higher in the seizure group, this difference did not reach statistical significance. Previous studies have demonstrated a transient increase in amino acid uptake during an interval of epileptic seizures, specifically uncontrollable seizures, and status epilepticus which also corresponded to structural MRI changes [16]. These changes were reversible concurrently with pathological EEG findings and postictal symptoms, which in some cases lasted up to eight weeks [16]. Our study cohort only comprised patients that had first-time epileptic seizures as the initial symptom of a DLGG and that were asymptomatic following initiation of ASM. The median interval from first epileptic seizure to PET acquisition was 13 days in our study cohort. Whether a single preceding seizure similarly affects [^18^F]FET uptake remains unknown. Notably, all patients in the present cohort were clinically seizure-free at PET acquisition. Nevertheless, as systematic EEG data were unavailable, an influence of subclinical epileptiform activity cannot be fully excluded. [^18^F]FET uptake in our cohort was confined to FLAIR-positive tumor volume, without extension into adjacent cortex or other perilesional regions. Thus, we did not observe the cortical ribbon-like amino acid uptake previously described in pronounced peri-ictal states, supporting that the measured FET signal predominantly reflected tumor-associated metabolic activity rather than seizure-related uptake in surrounding cortex.

Another study on temporal lobe DLGG correlated higher PET-positive tumor volume with seizure occurrence at initial diagnosis only in non-codeleted tumors [18]. In contrast, tracer uptake intensities were not linked to seizure presence at diagnosis but were associated with poorer postoperative seizure control at one year [18]. Notably, this study was restricted to temporal lobe DLGG, i.e. a brain region commonly known to be epileptogenic due to highly excitatory circuits, and primarily focused on postoperative seizure outcome. Our study cohort includes all supratentorial DLGG, representing a heterogenous cohort with variable susceptibility to seizure generation across cortical regions. Although our data show a difference in BTV this did not reach statistical significance. This could be due to the relatively small number of patients, specifically with no seizures.

In DLGG, tumors located in the frontal and temporal lobes, specifically close to functional areas are associated with an increased risk of epileptic seizures [2]. However, since DLGG are more commonly located close to or within eloquent brain structures which has been identified as an independent risk factor for the development of epileptic seizures, there is a natural bias [2]. This is reflected in our data to some extent, with a higher prevalence of tumors in the frontal lobe. To adjust for this type of bias, we compared the prevalence of [^18^F]FET uptake in regions known to be highly epileptogenic, i.e. frontal, insular and temporal lobes, with the prevalence of [^18^F]FET uptake in the remaining brain areas and did not see a statistically significant difference, thereby excluding that the demonstrated correlation simply reflects an increased prevalence of [^18^F]FET uptake in epileptogenic areas. Increased amino acid uptake in the context of tumor-associated epilepsy is not restricted to diffuse gliomas. Kasper et al. reported increased [^18^F]FET uptake in approximately two-thirds of patients with long-term epilepsy-associated glioneuronal tumors, demonstrating that substantial [^18^F]FET accumulation may also occur in typically low-grade epileptogenic lesions [19]. Similarly, Alkonyi et al. observed increased AMT uptake in the majority of epileptogenic temporal lobe dysembryoplastic neuroepithelial tumors (DNETs) [20]. Together with our findings, these observations suggest that increased amino acid uptake may represent a broader feature of metabolically active epileptogenic tumors rather than a phenomenon specific to IDH-mutant DLGG or [^18^F]FET PET.

The uptake of [^18^F]FET is primarily facilitated by the amino acid transporter LAT1 which is significantly higher expressed in glioma cells. The same study that demonstrated transient increase in amino acid uptake during seizure activity, also immunohistochemically confirmed increased upregulation of LAT1, LAT2 and CD98 transporters in neuronal and endothelial cells [16]. Although these data demonstrate upregulation of amino acid transporters in (sub)acutely seizure-affected brain, the overtime change in tumorous tissue remains unclear. Dysregulated glutamate signaling constitutes a key shared mechanisms of tumorigenesis and epileptogenesis with elevated glutamate levels promoting both neuronal hyperexcitability and tumor growth [4, 5]. Elevated glutamate levels are primarily mediated by the x_c_-cysteine glutamate transporter (xCT) [4, 21]. LAT1 and xCT are linked in the metabolic activity of glioma. xCT facilitates increased extracellular glutamate levels which is then converted in glutamine and transported back into the cell [21]. LAT1 is an antiporter, which uses glutamine in exchange for Leucin. High glutamate levels might, therefore, indirectly result in increased LAT1 activity, leading to increased uptake in [^18^F]FET PET. Furthermore, mTOR activation, which has also been identified as a mechanism in GRE, is dependent on essential amino acids, such as Leucin, which is transported via LAT1 [22]. Therefore, there is a biological rationale for generally higher LAT1 activity in the seizure group which also reflects in our data with higher TBR_max_ and TBR_mean_ values. This study cohort only included patients that were seizure-free following ASM initiation, therefore, this effect is independent of persisting seizure activity.

### Limitations

Several limitations should be acknowledged. Firstly, dynamic PET parameters were unavailable for most patients. Therefore, a more detailed characterization of [^18^F]FET uptake kinetics was not possible. Secondly, the observed association between [^18^F]FET uptake and preoperative seizures does not establish causality, nor can this data determine the direction of the relationship or its underlying mechanisms. Thirdly, the potential influence of ASM on [^18^F]FET uptake remains unclear. Although ASM has been reported to affect cerebral metabolism as measured by [¹⁸F]FDG PET, its potential impact on [^18^F]FET uptake is still unknown.

## Conclusion

This exploratory study has demonstrated that [^18^F]FET uptake is independently associated with GRE in patients with DLGG. One possible explanation for this is LAT1’s indirect link to the glutamine-glutamate cycle and its involvement in the mTOR activation, two known mechanisms in GRE. Further translational studies exploring the link between mechanisms of amino acid tracer uptake and GRE could give more insight into the pathophysiological mechanisms.

## Data Availability

No datasets were generated or analysed during the current study.
